# *Naja naja atra* Venom Protects against Manifestations of Systemic Lupus Erythematosus in MRL/lpr Mice

**DOI:** 10.1155/2014/969482

**Published:** 2014-06-30

**Authors:** Jiali Zhu, Kui Cui, Jianqun Kou, Shuzhi Wang, Yinli Xu, Zhihui Ding, Rong Han, Zhenghong Qin

**Affiliations:** Department of Pharmacology and Laboratory of Aging and Nervous Diseases, Jiangsu Key Laboratory of Translational Research and Therapy for Neuro-Psycho-Diseases, Soochow University School of Pharmaceutical Science, Suzhou 215123, China

## Abstract

Systemic lupus erythematosus (SLE) is an autoimmune disease and effective therapy for this pathology is currently unavailable. We previously reported that oral administration of *Naja naja atra* venom (NNAV) had anti-inflammatory and immune regulatory actions. We speculated that NNAV may have therapeutic effects in MRL/lpr SLE mice. Twelve-week-old MRL/lpr mice received oral administration of NNAV (20, 40, and 80 *μ*g/kg) or *Tripterygium wilfordii polyglycosidium* (10 mg/kg) daily for 16 weeks. The effects of NNAV on SLE manifestations, including skin erythema, proteinuria, and anxiety-like behaviors, were assessed with visual inspection and Multistix 8 SG strips and open field test, respectively. The pathology of spleen and kidney was examined with H&E staining. The changes in autoimmune antibodies and cytokines were determined with ELISA kits. The results showed that NNAV protected against the manifestation of SLE, including skin erythema and proteinuria. In addition, although no apparent histological change was found in liver and heart in MRL/lpr SLE mice, NNAV reduced the levels of glutamate pyruvate transaminase and creatine kinase. Furthermore, NNAV increased serum C3 and reduced concentrations of circulating globulin, anti-dsDNA antibody, and inflammatory cytokines IL-6 and TNF-*α*. NNAV also reduced lymphadenopathy and renal injury. These results suggest that NNAV may have therapeutic values in the treatment of SLE by inhibiting autoimmune responses.

## 1. Introduction

Systemic lupus erythematosus (SLE) is an autoimmune disease typified by the presence of autoantibodies and the deposition of immune complexes in multiple organs, including the skin, kidneys, heart, and joints, resulting in inflammation and tissue damage [1, 2].

Spontaneous SLE animal models, such as NZB×NZW F1, NBZ, BXSB, and MRL/lpr mouse strains, have been widely used for studying pathogenic mechanisms and experimental therapy [3]. They are characterized by lymphoid hyperplasia, B-cell hyperactivity, autoantibodies, presence of circulating immune complexes, complement activation, and renal injury [4]. The MRL/lpr especially has some advantages in the study of SLE. Firstly, the replication of lupus-like syndromes, such as serum levels of IgG [5], and psychiatric disorders [6] are more severe in female than that in male mice; this sex bias is common in human SLE (about a 9 : 1 female to male ratio) [7]. Secondly, its molecular defect in Fas gene can cause excessive B-cell activation mimicking the pathology in human SLE [8–10]. Due to these reasons MRL/lpr SLE mice were used in the present study.

Anti-inflammatory and immunosuppressive drugs such as glucocorticoids and cyclophosphamide are frequently used for the treatment of SLE in clinic, but these drugs have severe side effects [11–13].* Naja naja atra *venom (NNAV) is a complex mixture of bioactive peptides and proteins, including cobra venom factor, cardiotoxins, and neurotoxins [14]. Some reports have documented that NNAV has the anti-inflammatory effects [15, 16]. Recent studies showed that NNAV or its component cobratoxin could be used for the treatment of rheumatoid arthritis [16, 17], which is a common autoimmune disease. NNAV also produced a protective effect on acute and chronic nephropathy [15], which indicated that NNAV might have a potential for the treatment of lupus nephritis. It was reported that after oral administration of NNAV (30 and 100 *μ*g/kg) to mice for a year, the age related problems such as depilation, injury, and deterioration of various organs and inflammatory reaction were lightened in NNAV treated mice [18]. Long-term toxicity studies showed that the cobra venom had no influence on the routine blood test and vital organs, including heart, liver, spleen, lung, and kidney in normal animals [19]. The therapeutic doses of NNAV for anti-inflammatory and autoimmune diseases (20–200 *μ*g/kg) are far below its toxic doses (LD50 by oral was about 120 mg/kg, unpublished observations). These studies suggest that NNAV could be a valuable therapeutic alternative medicine for the treatment of immunological disorders. The purpose of the present study was to test the hypothesis that NNAV may have therapeutic values in treating MRL/lpr SLE mice.

## 2. Materials and Methods

### 2.1. Animals

Fifty female MRL/lpr mice aged 12 weeks (35 ± 3 g) were supplied by the Shanghai Slac Laboratory Animal Co., Ltd. and housed in a climatically controlled room (temperature 20 ± 2°C; humidity 50 ± 5%). However, the normal control mice MRL+/+ mice were not available in China and thus have been omitted. All experimental procedures were conducted according to the NIH Guidelines for the Care and Use of Laboratory Animals (NIH publication number 80–23, revised 1996) and were approved by the Committee on Animal Care and Use of Soochow University.

### 2.2. Drug Administration

NNAV was purchased from the Rainbow Snake Farm (Yujiang, Jiangxi Province, China). The snakes were house breed and venom was milked sterilely. Venom was dried with vacuum pump and was stored in freezer (−20°C). NNAV was heated to 100°C for 10 min and slowly cooled down to allow restoring nature conformation and biological activities with reduced toxicity before use [20]. NNAV solution was stored at 4°C and fresh solution was prepared every three days.

In the first experiment, six MRL/lpr mice were divided into three groups: model and NNAV (30, 100 *μ*g/kg), respectively. All mice were administrated for 16 weeks; the photographs on skin condition and the proteinuria were measured during the experiment.

In the second experiment, because* Tripterygium Wilfordii polyglycosidium* (TWP; Jiangsu Meitong Pharmaceutical Co., Ltd., China) has been widely used for the treatment of SLE disease [21–23], it was included in the study as a positive control. Fifty MRL/lpr mice were randomly divided into five groups: model, TWP (10 mg/kg), and NNAV (20, 40, and 80 *μ*g/kg), respectively. All mice received daily oral administration of drugs for 16 weeks and control mice received vehicle instead.

### 2.3. Detection of Proteinuria

For determination of proteinuria, mice were placed in the metabolic chambers individually for 24 h for collection of urines. Proteinuria was examined with Multistix 8 SG strips (Global Biotech Co., Ltd., Guangzhou, China) and was graded on the following scales as manufacturer recommended: 0–5, where 0 ≤ 10 mg/dL, 1 = 11–30 mg/dL, 2 = 30–100 mg/dL, 3 = 100–300 mg/dL, 4 = 300–2,000 mg/dL, and 5 ≥ 2,000 mg/dL.

### 2.4. Inner Open Field Test

The anxiety-like behaviors were determined with inner open field test instruments (JL Behv-LAG-4; Jiliang Software Technology Co., Ltd., Shanghai, China). Each mouse was gently moved to the center of the chamber, and the pattern of the movement of mouse was recorded for 30 min. At the end of each session, the chamber was cleaned. All the data were analyzed with a DigBehv animal behavior analysis system. The parameters used for analysis included total travel distance and central travel distance.

### 2.5. Photograph Acquisition

Allphotographs were taken with Olympus digital camera (C5050Z; Olympus, Tokyo, Japan) with a resolution of 72 dpi/inch. The graphs on skin damage in the pilot study were taken at the age of 24 weeks when the skin lesions in MRL/lpr mice appeared.

### 2.6. Blood Biochemistry Examination

At the end of the study, blood was collected from the eyeballs and centrifuged at 3000 rpm for 10 min; serum was separated and stored at −80°C until use. Blood biochemical indexes of serum were measured with an automatic biochemical analyzer (GRT-3002; Gelite Technology Co., Ltd., Jinan, China).

### 2.7. ELISA Assay

The concentrations of IgG anti-dsDNA, IL-6, TNF-*α*, and complement C3 in serum were determined using appropriate commercial ELISA kits (Hushang Biotech Co., Ltd., Shanghai, China) following the manufacturer's protocols. The absorbance of each sample was read at 450 nm with a microplate spectrophotometer (BioTek, VT, USA).

### 2.8. Histological Examination

Spleens and kidneys were dissected immediately after mice were killed. Tissues were fixed in 10% PBS-buffered formalin for 24 h and then embedded in paraffin wax. Sections at 5 *μ*m were stained with hematoxylin and eosin (H&E) and examined with a light microscopy (Olympus, Tokyo, Japan). The areas of white pulps in spleen sections were calculated with Image-Pro Plus. The index of lupus nephritis was scored according to histopathologic appearance of kidney section [24]: 0 = normal, 1 = a little cell infiltration in the mesangium, 2 = a more cell infiltration in the mesangium, 3 = thickened basement membrane and glomerular lobular formation, and 4 = tubular casts, atrophy, glomerular crescent formation, and sclerosis.

### 2.9. Statistical Analysis

Data are presented as mean ± SD. Statistical analyses were performed using Graph Pad Prism 6.0 software. A two-factor ANOVA was used to test for drug interactions. When a significant interaction was ensured, a one-way ANOVA with a Student-Newman-Keuls post hoc test was used to test differences between specific groups. Significance was considered when *P* < 0.05.

## 3. Results

General description: skin lesions with scab formation, hair loss, and proteinuria are common in MRL/lpr mice [25]. In our first experiment, the abnormal skin manifestation of MRL/lpr mice appeared at the age of 24 weeks, which was mainly displayed as red spots in head, neck, back, ears, and tail initially, became larger patches in two weeks, and then changed to scab and fell off. A robust hair loss occurred where red patches were observed. The skin of ear was severely damaged and deformed due to scars formation. The tail erosion and auricle broken were seen. It was noted that the skin lesions in MRL/lpr mice were significantly reduced by NNAV (30 and 100 *μ*g/kg) with no red patches or spots, no skin fester, and no apparent hair loss (see Supplementary Figure 1(a) of the Supplementary Material available online at http://dx.doi.org/10.1155/2014/969482). The proteinuria score could represent the kidney damage; NNAV (30 and 100 *μ*g/kg) also showed a protect effect on this morbid change (Supplementary Figure 1(b)). These preliminary results suggested that NNAV had a therapeutic effect in MRL/lpr mice. Thus a full-scale study was performed to confirm the therapeutic effects of NNAV in MRL/lpr mice.

The life span of MRL/lpr mice is about five to eight months [24]; we started our experiment at the age of 12 weeks, and the mice began to die at the age of 26 weeks. Because of the limitation of the quantity of mice, we ended our experiment at 28 weeks of age. The number of mice in each group was ten at the start of the study. The amount of survived mice in model and NNAV-treated 40 and 80 *μ*g/kg groups was eight at the end of the study. No death occurred in TWP- or NNAV-treated 20 *μ*g/kg group.

### 3.1. Effects of NNAV on Lymphadenopathy

**Figure 1:**
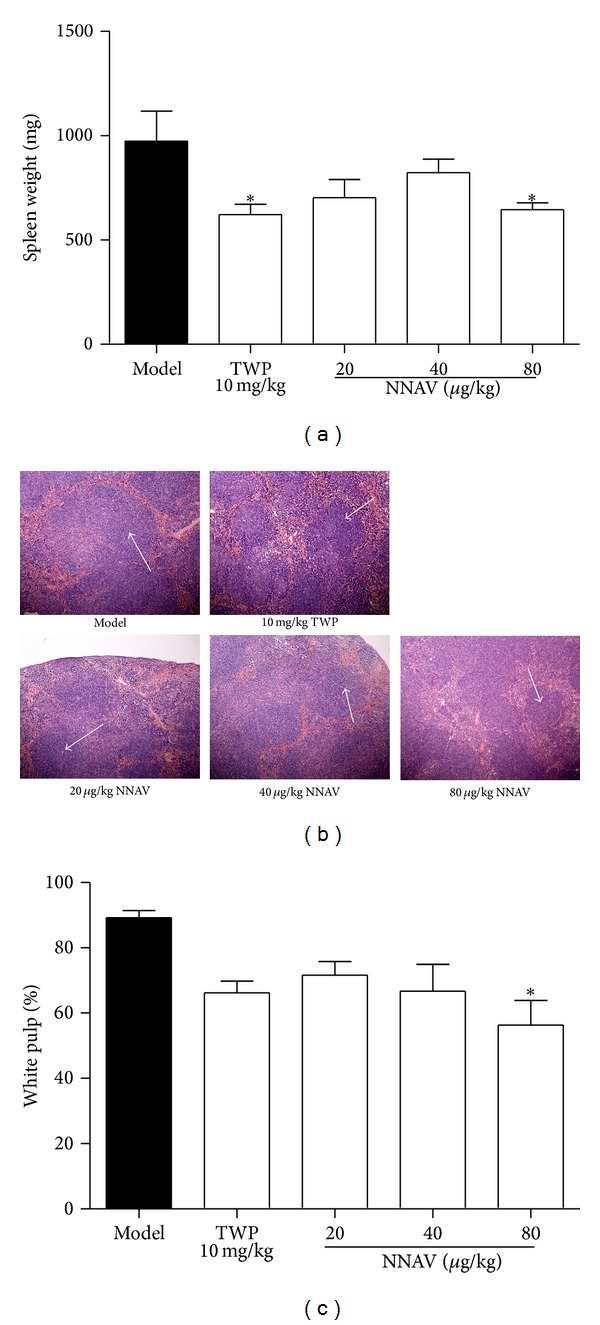
NNAV reduced the lymphadenopathy. After treatment of MRL/lpr mice with NNAV for 16 weeks, mice were killed and spleens were dissected. All spleen samples were weighed immediately (a). Data represent mean values ± SD from 8 to 10 mice per group. *P < 0.05 (versus model). Spleen sections (b) were stained with hematoxylin and eosin and observed with a microscopy at 100x magnification. Arrows: white pulp. The area of white pulp (c) was calculated with Image-Pro Plus. Data represent mean values ± SD from 4 mice per group. *P < 0.05 (versus model).

One of the most important indexes of SLE progression in MRL/lpr mice is lymphadenopathy [26]: the spleen weight could increase to 500 mg (18 weeks)–1,000 mg (24 weeks), almost ten times larger than normal mice [27–29]. In the present study, the spleen enlargement in the model mouse was robust (975 ± 321 mg) at the age of 28 weeks. NNAV significantly reduced the spleen weight (Figure 1(a)), especially at the dose of 80 *μ*g/kg compared to model group (*P* < 0.05). The composition of white pulp is T and B lymphocytes. The altered Fas gene resulted in failure in programmed cell death of lymphocytes in the spleen. The enlarged spleen is mainly due to the excessive proliferation of T and B lymphocytes. As shown in Figure 1(b), the white pulp almost occupied the whole field of vision in model group, which indicated the excessive proliferation of lymphocytes. When NNAV was given, the diffuse hyperplasia of white pulp in spleen was inhibited (Figure 1(c)).

### 3.2. Effects of NNAV on Autoimmune-Induced Organ Damage

#### 3.2.1. Skin Damage

The skin damage in the second experiment was mild compared to the first experiment. The skin pathology was only found in the neck. We observed that the skin conditions were also improved in NNAV-treated group; however, there was no obvious improvement in skin damage in TWP-treated group compared with model group. As the skin manifestation was not robust, the photograph was not taken during the second experiment.

#### 3.2.2. Proteinuria

**Figure 2:**
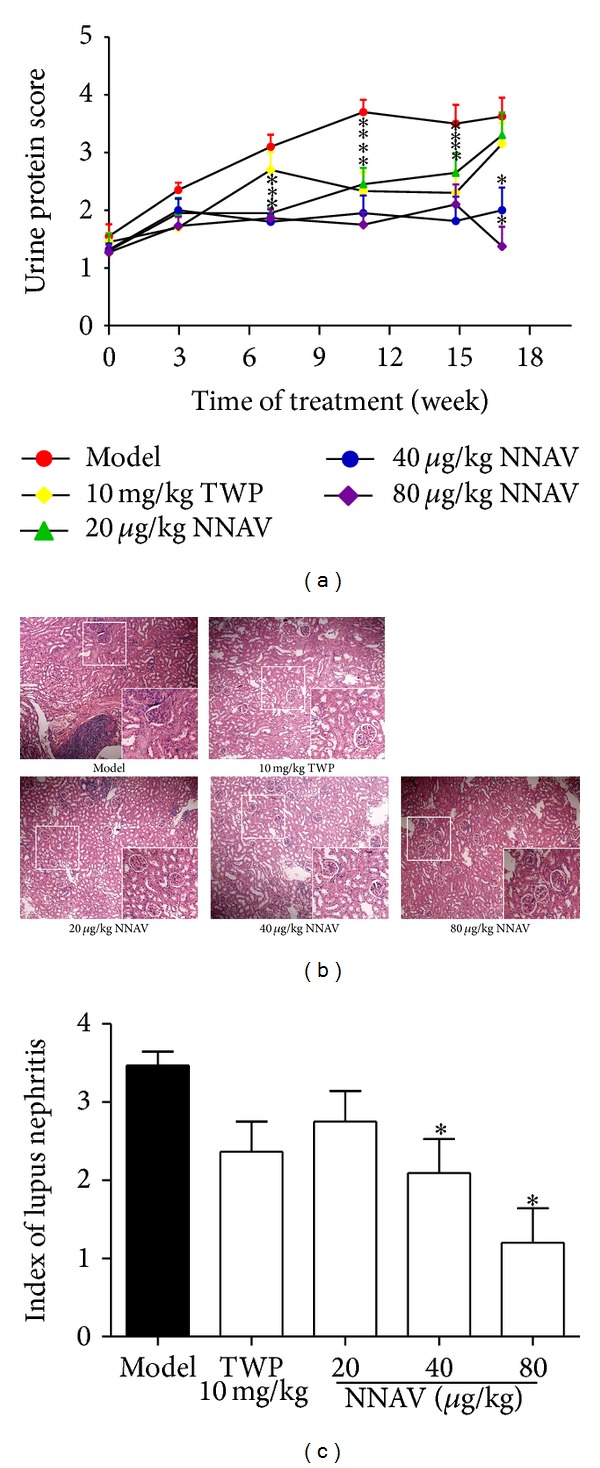
NNAV reduced the proteinuria. During the 16 weeks of NNAV administration, proteinuria (a) in each group was measured with Multistix 8 SG strips following the manufacturer's instructions, where 0 ≤ 10 mg/dL, 1 = 11–30 mg/dL, 2 = 30–100 mg/dL, 3 = 100–300 mg/dL, 4 = 300–2,000 mg/dL, and 5 ≥ 2,000 mg/dL. Data represent mean values ± SD from 8 to 10 mice per group. *P < 0.05 (versus model). At the end of the study, kidneys from each mouse were collected and the kidney sections (b) were stained with hematoxylin and eosin and observed with a microscopy at 100x magnification. The index of lupus nephritis (c) was scored according to histopathologic appearance of kidney section. Data represent mean values ± SD from 4 mice per group. *P < 0.05 (versus model).

**Figure 3:**
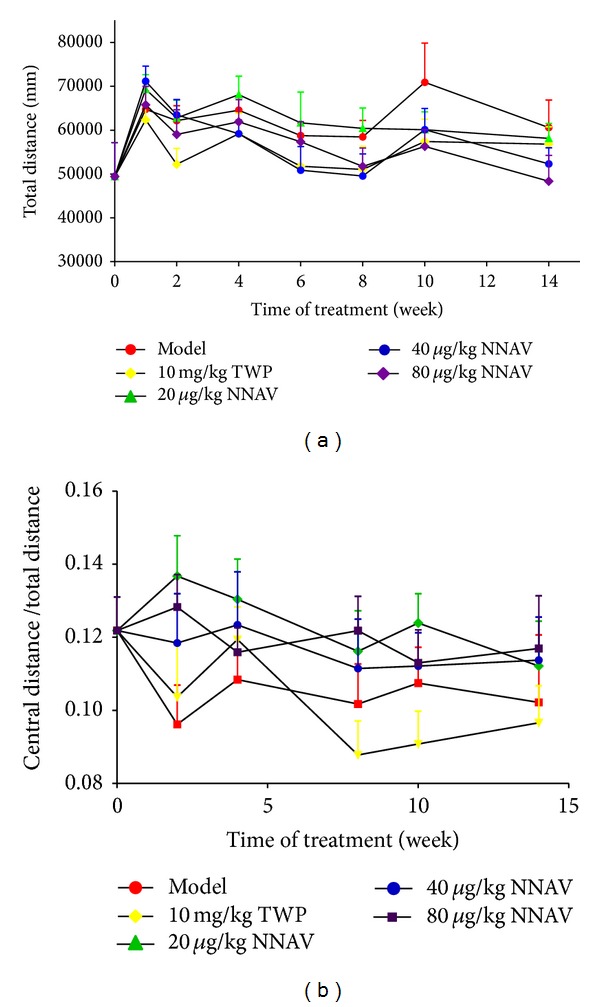
The effects of NNAV anxiety-like behaviors. Inner open field test was taken during the experiment. And the test time for each mouse was 30 min. The total travel distance (a) and central travel distance were determined with the DigBehv animal behavior analysis system. The ratio of central distance to total distance was calculated (b). Data represent mean values ± SD from 8 to 10 mice per group.

The development of lupus-like renal disease is a major feature in MRL/lpr mice [30]. And the renal dysfunction could finally lead to death of mice in the late stage of the disease [31]. Proteinuria is a major symptom to indicate the progression of renal disease. As presented in Figure 3(a), the renal disease already existed in MRL/lpr mice at the age of 12 weeks (urine protein score > 1), and the concentration of protein in urine continued to increase in model mice thereafter. In contrast, after 7 weeks of administration of NNAV, the levels of proteinuria began to decrease compared to model group, and the significant decrease in TWP group has appeared after 11 weeks of drug administration (Figure 2(a)). In consistency with this result, the nephritic glomerulus in the model group displayed cell infiltration with crescent formation. In TWP- or NNAV-treated MRL/lpr mice, the structure of the glomerulus appeared nearly normal with less infiltration of cells or membranoproliferation (Figure 2(b)). The calculated index of lupus nephritis in Figure 2(c) also showed the improvement of nephropathy.

#### 3.2.3. Anxiety-Like Behaviors

It is estimated that the prevalence of neuropsychiatric systemic-SLE in human is about 14% to 75% [32–34]. The evolvement of SLE in MRL/lpr mice is also accompanied by behavioral abnormalities, such as depression and anxiety-like behaviors [35, 36]. In order to explore whether NNAV had ameliorative effect on the anxiety-like behaviors in MRL/lpr mice, the inner open field test was performed [36]. The reduced exploration in the central field reflects anxiety; meanwhile, the total distance traveled represents the locomotor activity. In the present study, we found that model mice were very agitated in response to rout animal handling. Mice in model group start to jump when we attempted to grab them, whereas NNAV-treated mice were much easy to handle with much gentle response. As shown in Figure 3, NNAV reduced the total distance with a less reduction in the central distance and thus slightly increased the ratio of central travel distance to total travel distance, indicating that NNAV had the tendency to produce an antianxiety effect.

#### 3.2.4. Others

**Figure 4:**
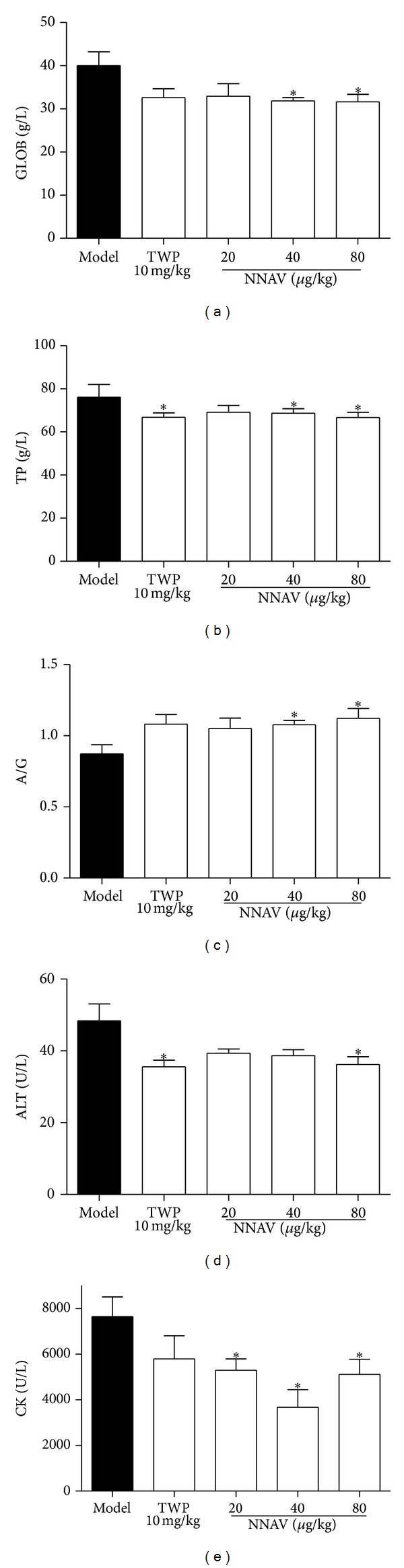
NNAV improved the ratio of albumin/globulin and suppressed the biochemical index of liver and heart injury. The serum was collected at the end of the study and the concentrations of globulin (a) and total protein (b) were measured with automatic biochemical analyzer, and ratio of albumin/globulin (c) was calculated. ALT (d) and CK (e) concentrations were measured with automatic biochemical analyzer. Data represent mean values ± SD from 8 to 10 mice per group. *P < 0.05 (versus model).

**Figure 5:**
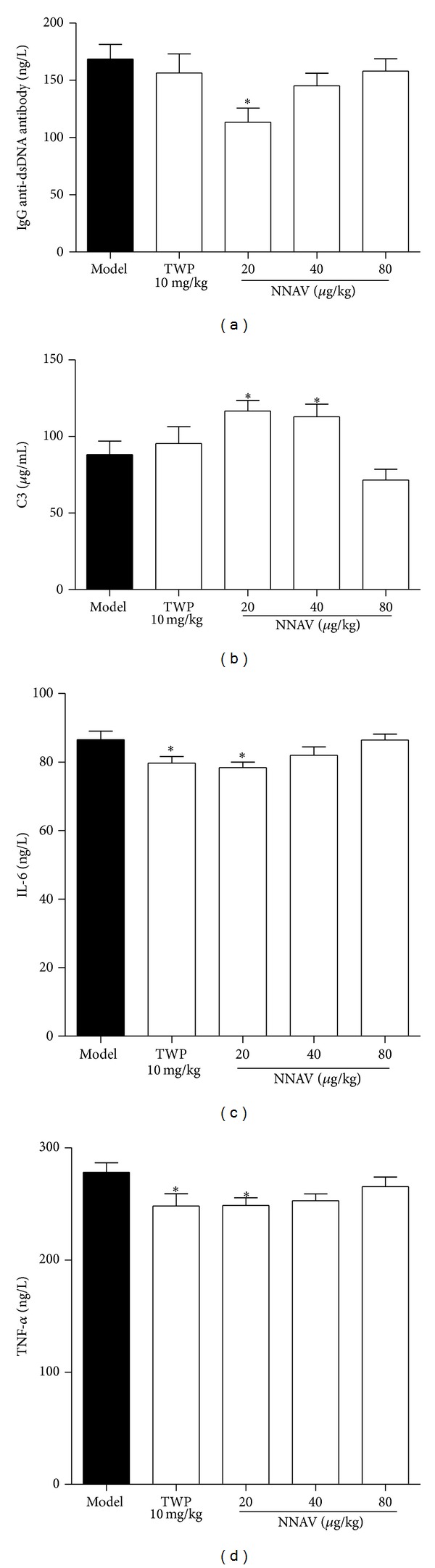
NNAV suppressed autoimmune activity. After 16 weeks of NNAV administration, the serum was collected for the determination of IgG anti-dsDNA (a), C3 (b), IL-6 (c), and TNF-*α* (d) using ELISA kit. Data represent mean values ± SD from 8 to 10 mice per group. *P < 0.05 (versus model).

SLE is a systemic autoimmune disease; thus many organs and tissues are attacked by the immune system. The high concentration of globulin in serum indicates the overactivity of the immune system. As shown in Figure 5, the globulin concentration was increased in model mice (Figure 4(a)), resulting in the upregulation of the levels of total proteins (Figure 4(b)) and the inversion of the albumin/globulin (A/G) ratio (Figure 4(c)). NNAV showed a dose-dependent improvement on these pathological changes. In addition, although no abnormal histology was found in liver and heart, the levels of glutamate pyruvate transaminase (ALT) (Figure 4(d)) and creatine kinase (CK) (Figure 4(e)) in serum were also reduced by TWP and NNAV, indicating that the injury of liver and heart was probably ameliorated.

#### 3.2.5. Effects of NNAV on Autoimmune Activity

Several autoimmune and inflammatory indices were measured in serum to probe the potential effects of NNAV on immune activity. Autoantibody production is a sensitive clinical measurement for SLE. As shown in Figure 5(a), NNAV inhibited the production of IgG anti-dsDNA, especially at the dose of 20 *μ*g/kg (*P* < 0.05). The low levels of C3 in serum in model mice (Figure 5(b)) were recovered in NNAV-treated mice, particularly at the doses of 20 *μ*g/kg and 40 *μ*g/kg (*P* < 0.05). TWP and NNAV at dose of 20 *μ*g/kg or 40 *μ*g/kg could significantly inhibit the production of inflammatory cytokines IL-6 (Figure 5(c)) and TNF-*α* (Figure 5(d)) compared to model group (*P* < 0.05).

## 4. Discussion

Systemic lupus erythematosus (SLE) is an autoimmune disease; immunosuppressive and anti-inflammatory agents such as cyclosporine A and steroid hormones are used in clinic for the prevention of SLE [37]. However, SLE patients not only die from renal failure but also die from the immunosupppression induced opportunistic infection and cancer. Belimumab, targets on B-cells [38], was approved by US Food and Drug Administration (FDA) for the treatment of SLE recently, which opened a new sight of SLE therapy. Just like belimumab, which aimed at specific target that mediates the SLE, previous study in our laboratory showed that oral administration of NNAV had the ability of inhibiting CD8 and Th17 cell differentiation [39], which mainly mediate inflammation in autoimmune disease [40, 41]. Thus NNAV may have the potential for the treatment of SLE. The present study found that oral administration of NNAV inhibited the manifestation of SLE in MRL/lpr mice, including lymphadenopathy, skin damage, proteinuria, inflammatory cytokines, and autoimmune damage of immune organs.

MRL/lpr mice model is one of the commonly used spontaneous SLE models whose symptoms are similar to human SLE, including immune complex-induced organ injury [3]. It is mainly due to the recessive mutations of the Fas gene which is associated with spontaneous programmed cell death [42]. Thus, the hyperactive lymphocyte proliferation leads to T- and B-cell overproduction and systemic lymph organ enlargement and finally accelerates the process of the autoimmune response [42]. Chronic treatment of MRL/lpr mice with NNAV protected the lymphoid hyperplasia of spleen controlled by the Fas gene in these mice (Figure 1). The inhibitory effects of NNAV on autoimmune antibody production in MRL/lpr mice (Figures 4(a) and 5(a)) might be directly due to the suppression on T- or B-cell overt proliferation triggered by mutation of Fas gene.

It is common that SLE patients have high levels of total serum protein and globulin and low levels of the albumin/globulin ratio [43–45]. In the present study, the MRL/lpr mice showed a rise in globulin production and resulted in the reversal ratio of the albumin/globulin. When NNAV was administrated, this alteration was ameliorated (Figures 4(a)–4(c)). It is well demonstrated that the globulin is homogeneous with antibody [44]. In the progression of the SLE, the antibody production increased with a preferential increase in varieties of autoantibodies, including antinuclear antibody, anti-double-stranded (ds) DNA, and Sm protein [46]. Among these, the anti-dsDNA antibodies are very specific for SLE [47]. IL-6 has been shown to be important in the generation of SLE disease associated antibodies identifying dsDNA [48, 49]. The results in our research showed that oral administration of NNAV could reduce the concentration of anti-dsDNA in serum (Figure 5(a)), and this effect may be due to the inhibition of the IL-6 production (Figure 5(c)).

The stimulatory effect of immune complexes (ICs) on the complement system and the consumption of complement are two features in active phase of SLE [23, 50]. The low concentration of complement in serum indirectly indicates the augmentation of ICs in serum. Defective clearance of ICs, together with autoantibodies against DNA and nuclear proteins, contributes to SLE manifestations associated with deposition of ICs in skin, kidney, heart, and other organs [51]. The organ affected by SLE mainly displayed nonspecific inflammation. TNF-*α* is imperatively involved in the pathogenesis of this disease [52]. NNAV increased the circling component C3 (Figure 5(b)) and decreased the inflammatory cytokine TNF-*α* (Figure 5(d)) production. This demonstrated that NNAV could inhibit autoimmune damage in MRL/lpr mice, and the curing effect may owe to the reduction in CS and the suppression on TNF-*α* secretion.

NNAV suppressed the skin damage spreading (Supplementary Figure 1(a)), depressed the urine protein output, and improved the renal glomerulus histology (Figure 2). Daily administration of NNAV also significantly reduced the contents of ALT (Figure 4(d)) and CK (Figure 4(e)). Glutamate pyruvate transaminase (ALT) and creatine kinase (CK) in serum are used in the evaluation of liver and heart injury [53]. This indicated that NNAV prevented liver and heart morbid change as well.

The neuropsychiatric abnormalities were linked to the autoimmune response in central nerves system (CNS). The chemokines or inflammatory cytokines such as IL-6 and TNF-*α* production in MRL/lpr mice may be involved in anxiety behaviors [54–56]. In our study, NNAV showed a potential to reduce the anxiety-like behavior (Figure 3), and these may be due to the inhibitory effect on the secretion of IL-6 and TNF-*α* (Figures 5(c) and 5(d)). However, as NNAV may not easily get into CNS, thus the effects of NNAV on CNS may be limited.

The molecular target involved in immune modulation by NNAV is currently unknown. The main active component for analgesic effects of NNAV is neurotoxin [57]. The mechanism of analgesic effects of neurotoxin involves nAch and mAch receptors [57]. We speculate that the active component involved in immune regulation may also be mainly produced by neurotoxin and is mediated by cholinergic mechanisms.

The major pitfall in the present study is the lack of a normal control. The normal control was not included in our study as the MRL+/+ mice were not available in China. The mouse model of MRL/lpr was developed by Murphy ED at the year of 1978 by crossbreeding of four species of mice. We found that the normal control was also not integrant in some published studies [58–60]. With no normal proper normal control in our studies, all results can only be compared with presymptomatic levels to demonstrate the effectiveness of NNAV treatment.

Another point should be noticed is the poor dose-effect relationship of NNAV. The dose-effect relationship in our study is complicated, and the exact reason for this phenomenon is not clear. NNAV is a mixture of many different components which exert different activities and their actions may be synergistic or opposing to each other. This may be the reason why NNAV displays not only nonlinear dose-effect relationship but also no consistent dose-effect relationship among the various physiological indexes.

In conclusion, NNAV could ameliorate SLE symptoms, improve lymphadenopathy, and reduce autoimmune activity and inflammation. Thus, NNAV could be a valuable alternative medicine for the effective treatment of SLE disease.

## Supplementary Material

In the pilot study, six MRL/lpr mice were divided into three groups: model and NNAV (30, 100 *µ*g/kg), respectively. All mice were administrated for 16 weeks. The photographs on skin condition were taken with Olympus digtal camera (C5050Z; Olymus, Tokyo, Japan) at the age of 24 weeks when the skin lesions appeared, and the proteinuria were measured with Multistix8 strips (Global Biotech Co., Ltd., Guangzhou, China) during the experiment.
