# Supplementary figures and images for: Naja naja atra Venom Protects against Manifestations of Systemic Lupus Erythematosus in MRL/lpr Mice
Source: Evid Based Complement Alternat Med. 2014 Jun 30;2014:969482. doi: 10.1155/2014/969482 (PMC4100264; doi:10.1155/2014/969482)

S-figure1A

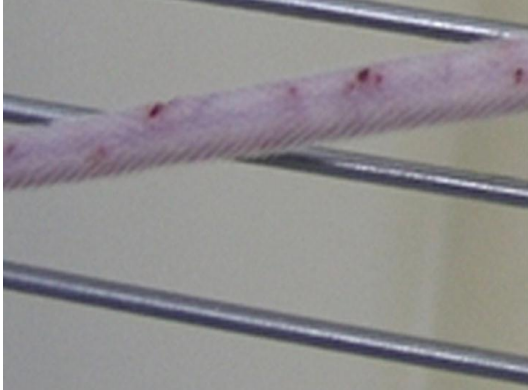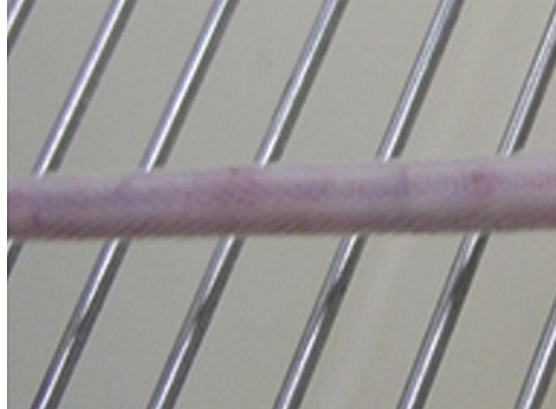

tail

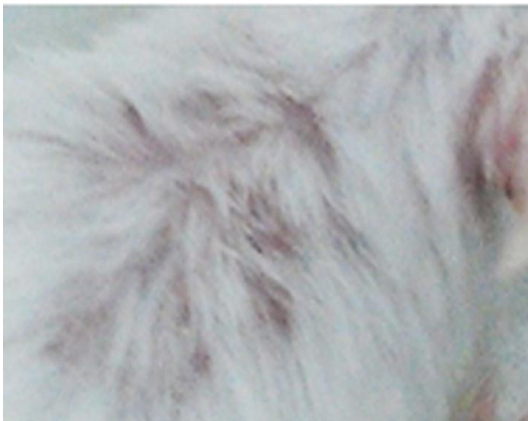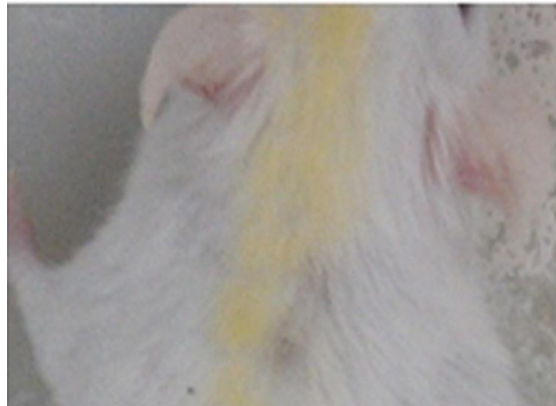

neck

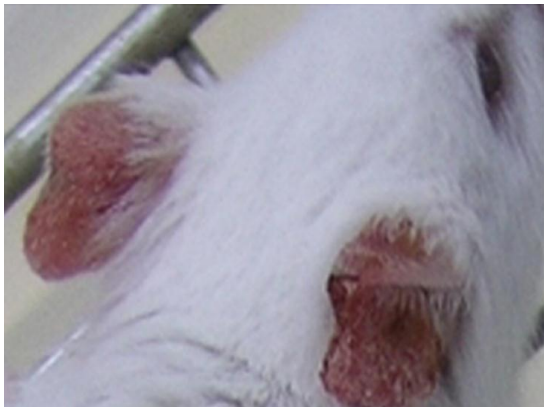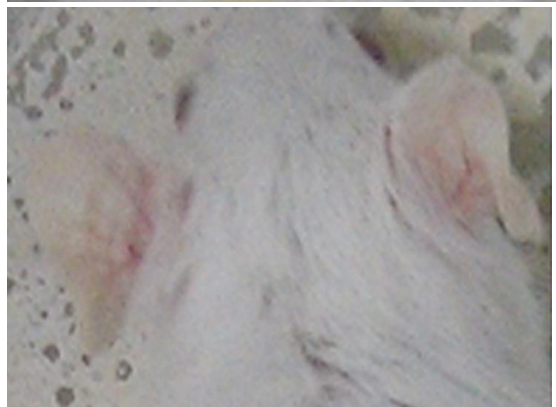

ear

model

NNAV

S-figure1B

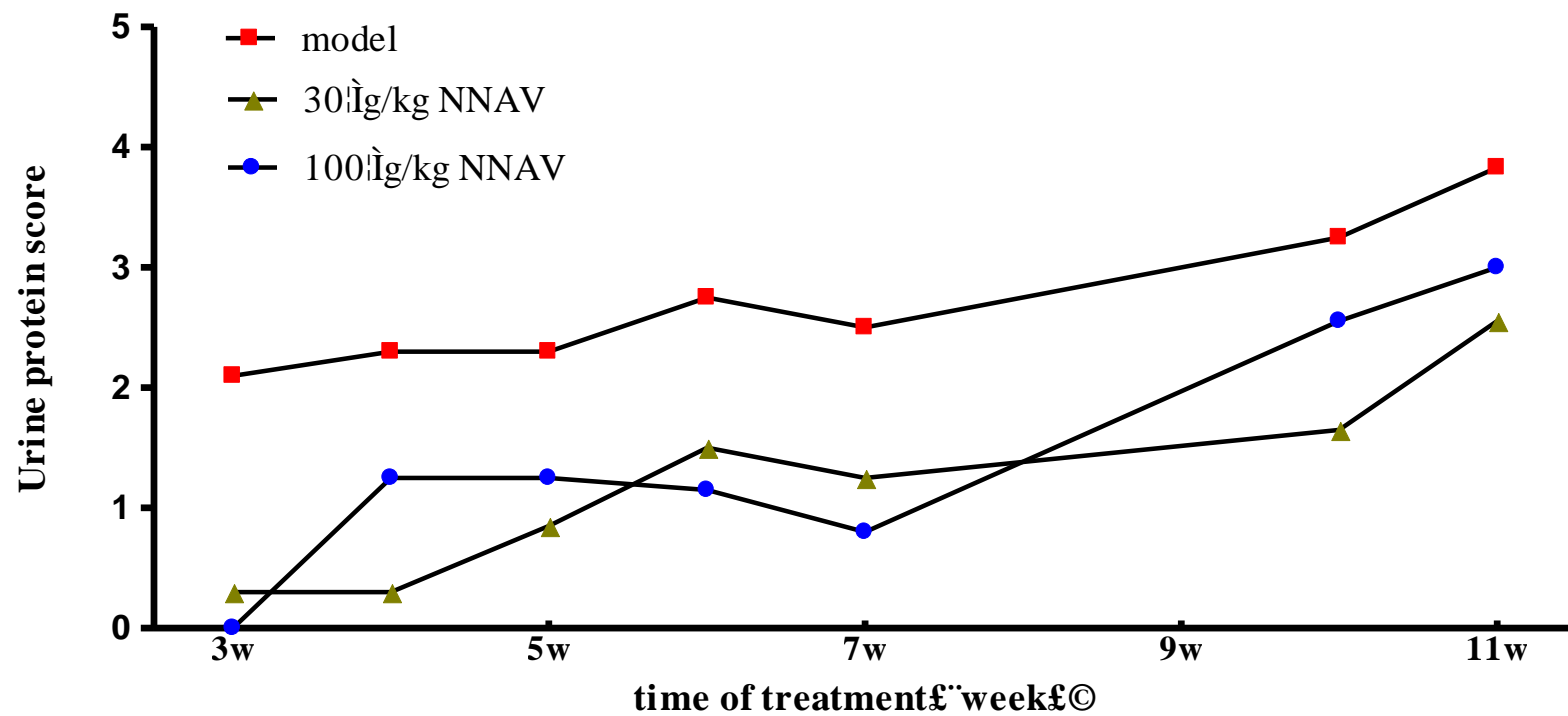

Supplement: Supplementary file 1 — In the pilot study, six MRL/lpr mice were divided into three groups: model and NNAV (30, 100 µg/kg), respectively. All mice were administrated for 16 weeks. The photographs on skin condition were taken with Olympus digtal camera (C5050Z; Olymus, Tokyo, Japan) at the age of 24 weeks when the skin lesions appeared, and the proteinuria were measured with Multistix8 strips (Global Biotech Co., Ltd., Guangzhou, China) during the experiment. [file 969482.f1.pdf]
